# Climate-Driven Range Shift of the Medicinal Herb *Epimedium sagittatum*: An Optimized MaxEnt Projection for China

**DOI:** 10.3390/biology15141103

**Published:** 2026-07-08

**Authors:** Jun Luo, Suhang Li, Fuyuan Huang, Qiong Yang, Yangzhou Xiang, Ying Liu

**Affiliations:** 1 Guizhou University of Traditional Chinese Medicine, Guiyang 550025, China; 2 School of Geography and Resources, Guizhou Education University, Guiyang 550018, China; 3 School of Biological Sciences, Guizhou Education University, Guiyang 550018, China

**Keywords:** *Epimedium sagittatum*, climate change, MaxEnt model, parameter optimization, conservation planning

## Abstract

*Epimedium sagittatum* is an important understory shade-tolerant medicinal plant native to China. However, assessments of its responses to climate change under multiple scenarios and time periods remain insufficient, particularly regarding the mitigation of model overfitting and the quantitative disentanglement of hydrothermal constraints. In this study, based on 269 valid occurrence records and 24 initial environmental variables (reduced to 13 after collinearity screening), we employed a parameter-optimized MaxEnt 3.4.4 model (RM = 3.5, FC = QHP) coupled with the BCC-CSM2-MR climate model to project the dynamics of potential suitable habitats under current (1970–2000) and future (2050s, 2070s, 2090s) scenarios (SSP126, SSP370, SSP585). The results showed that the optimized model substantially reduced the risk of overfitting compared with the default parameters (ΔAICc dropped from 82.16 to 0) and achieved an AUC of 0.934. Precipitation of the Driest Quarter (Bio14, 53.4% contribution) and Minimum Temperature of the Coldest Month (Bio6, 20.5% contribution) were identified as the dominant factors governing the species’ distribution, with optimum conditions of ≥1.58 mm and −8.34 °C to 13.61 °C, respectively. Under future climate scenarios, the centroid of suitable habitats shifted progressively southwestward, with a cumulative displacement of approximately 127.47 km under SSP585, and water availability, rather than temperature, dominated the direction of this shift. Under the high-emission scenario, suitable areas exhibited a spatial reorganization characterized by westward expansion and eastward contraction. Based on these findings, this study proposes a three-tier planning framework comprising in situ conservation, climate-smart introduction, and assisted migration, providing a scientific basis for the conservation of germplasm resources and the spatial planning of artificial cultivation of *E. sagittatum*.

## Simple Summary

*Epimedium sagittatum*, a Chinese medicinal herb traditionally used for bone and kidney health, faces rapid wild population decline due to overharvesting, habitat fragmentation, and climate change. Using 269 verified field records and an advanced ecological model, we projected its future distribution under multiple climate scenarios. Two key constraints emerged: dry-season rainfall and winter cold extremes. Under the highest-emission pathway, suitable habitats were forecast to contract and shift southwestward by over 127 km by the 2090s, with severe losses in Southern China. In response, we recommend (1) protecting climatically stable hill regions in Jiangxi, Zhejiang, and Hunan; (2) developing climate-adapted cultivation in montane Sichuan, Chongqing, and southern Shaanxi; and (3) systematically banking seeds from vulnerable southern populations to preserve genetic diversity. This integrated strategy provides a practical roadmap for sustaining *E. sagittatum* as environmental conditions continue to change.

## 1. Introduction

Global climate change has substantially altered the geographic distribution patterns of plant species, manifesting as range contraction toward higher latitudes and elevations, increased population fragmentation, and elevated risks of local extinction [1,2,3]. Species distribution models (SDMs), which establish statistical relationships between species occurrence records and environmental predictors, have become a central tool for assessing the impacts of climate change on habitat suitability [4,5]. Among various SDMs, the MaxEnt model is widely used to predict suitable habitats for medicinal plants, owing to its ability to handle presence-only data, robustness to sample size variation, and strong predictive performance [6,7,8]. This model integrates the strengths of different modeling approaches, generating extrapolable predictions while appropriately capturing interpolation complexity, thereby improving modeling efficiency [9]. However, default parameter settings, specifically a regularization multiplier (RM) of 1 and a feature combination (FC) of LQHP, tend to induce overfitting [10]. Overfitting is characterized by inflated training AUC values coupled with poor generalization to test data, ultimately yielding ecologically unreliable distribution predictions [11,12]. Therefore, parameter optimization based on the Akaike Information Criterion (AICc) and the 10% training omission rate (OR.10) [13] has become an essential step to enhance the ecological interpretability and predictive reliability of MaxEnt models.

*Epimedium sagittatum* (Sieb.et Zucc.) Maxim. (*E. sagittatum*) is a perennial herb belonging to the genus *Epimedium* (Berberidaceae) and serves as one of the source plants for the traditional Chinese medicinal material “Yinyanghuo”. It exhibits a range of pharmacological activities, including kidney tonification, anti-osteoporosis activity, and neuroprotection [14,15,16,17]. Unlike the subjects of most existing species distribution modeling studies, such as alpine plants or invasive weeds, *E. sagittatum* is a shade-tolerant plant native to subtropical evergreen broadleaved forests. In recent years, owing to unsustainable harvesting and habitat fragmentation, wild populations of this species have declined sharply, leading to its designation as a locally protected species in multiple regions [18,19]. Concurrently, market demand for Epimedium herbs continues to grow, making artificial cultivation a primary pathway through which resource pressure can be alleviated [20,21,22]. However, problems such as low yield and quality degradation frequently arise from blind introduction, which fundamentally results from an insufficient understanding of the climatic and edaphic niche boundaries of *E. sagittatum*. Therefore, quantitatively identifying the dominant environmental factors that limit its geographic distribution and forecasting the dynamics of suitable habitats under climate change are of critical practical importance for developing scientifically sound domestication strategies and cultivation base layouts [23,24,25].

Although several studies have predicted suitable habitats for certain *Epimedium* species using MaxEnt model [26,27,28,29,30], a systematic, national-scale, multi-scenario, and multi-period assessment specifically targeting *E. sagittatum* remains lacking. These existing studies generally share several limitations: (1) they adopt default parameters without balancing model complexity against overfitting risk; (2) they lack systematic collinearity diagnostics among variables (|r| ≥ 0.7), which may lead to misidentification of dominant drivers; and (3) they often overlook the integrated regulatory roles of topography (e.g., slope, aspect), vegetation (NDVI), and human activities (human footprint index). For a shade-adapted understory plant like *E. sagittatum*, the soil moisture retention and light conditions modulated by slope and aspect may be particularly critical, yet these factors are frequently neglected in conventional modeling. Recently, the ENMeval package has been shown to markedly improve the generalization capacity and ecological explanatory power of MaxEnt by tuning the regularization multiplier (RM = 0.5–4) and feature combinations (FC, e.g., various combinations of L/Q/H/P/T), with the optimal model selected based on AICc and the 10% training omission rate (OR10) [13,31]. Moreover, the BCC-CSM2-MR climate model developed by the Beijing Climate Center demonstrates high simulation accuracy over the East Asian monsoon region [32,33]. When coupled with multiple representative concentration pathways, this model enables robust assessment of the spatiotemporal dynamics of plant suitable areas across different time periods. Nevertheless, this optimization framework has not yet been applied to the distribution modeling of *E. sagittatum*.

Based on the above considerations, this study focuses on *E. sagittatum* by integrating multi-source distribution records with four categories of environmental variables (climate, topography, vegetation, and human activity) and constructs a standardized MaxEnt modeling workflow in R. The objectives are threefold. First, we aimed to identify the environmental factors governing the species’ geographic distribution and quantify their ecological optimum ranges, with particular emphasis on hydrothermal constraints in understory habitats. Second, using the optimized MaxEnt model and the BCC-CSM2-MR climate model, we aimed to project potential suitable habitat distributions under current (1970–2020) and three future periods (2050s, 2070s, 2090s) across different climate scenarios (SSP126, SSP370, SSP585). Third, we sought to reveal the area dynamics, spatial pattern changes, and centroid migration trajectories of suitable habitats under climate change. Accordingly, we propose three hypotheses: (1) Precipitation of the Driest Quarter (Bio14) and Minimum Temperature of the Coldest Month (Bio6) are the core factors limiting the distribution of *E. sagittatum*; (2) future suitable habitats will shift northwestward; and (3) the high-emission scenario (SSP585) will cause significant range contraction and fragmentation. These findings are intended to provide a scientific basis for germplasm conservation, the selection of domestication sites, and climate-adaptive cultivation strategies for *E. sagittatum*.

## 2. Materials and Methods

### 2.1. Overview of the Modeling Workflow

To ensure reproducibility and provide a clear overview of the analytical pipeline, the entire modeling procedure comprised four sequential steps: (1) collection and quality-control of occurrence records, coupled with spatial thinning to reduce sampling bias; (2) multicollinearity screening of 24 candidate environmental variables (climate, topography, vegetation, and human activity) via Spearman correlation analysis, retaining variables with |r| < 0.7 and supplemented by initial MaxEnt contribution rates for correlated groups; (3) optimization of the MaxEnt model using the ENMeval package across a range of regularization multipliers (0.5–4.0) and nine feature combinations, with final model selection based on ΔAICc and the 10% training omission rate; and (4) projection of current and future habitat suitability under three SSP scenarios (SSP126, SSP370, SSP585) across three time periods (2050s, 2070s, 2090s), followed by spatial analyses of area dynamics, stability, and centroid migration.

### 2.2. Acquisition and Processing of Distribution Data

**Figure 1:**
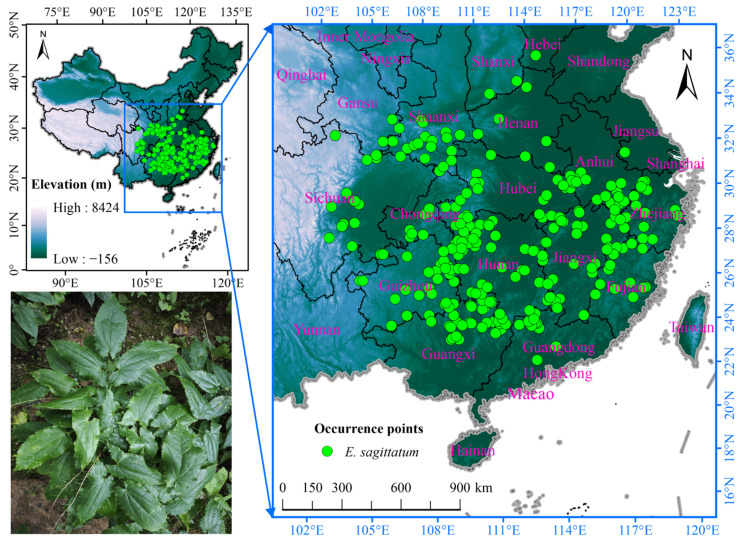
Distribution records of *E. sagittatum* in China.

Occurrence data of *E. sagittatum* in China were collected from public databases and literature: the Chinese Virtual Herbarium (CVH, https://www.cvh.ac.cn/, accessed on 12 May 2026), the Global Biodiversity Information Facility (GBIF, Available online: https://doi.org/10.15468/dl.8k6m8f, accessed on 17 May 2026), China National Knowledge Infrastructure (CNKI, https://www.cnki.net/, accessed on 18 May 2026), and Google Scholar (https://ac.scmor.com/, accessed on 19 May 2026). For records lacking coordinates but with detailed site descriptions, geographic coordinates were retrieved using the Jingweidu coordinate query tool (http://jingweidu.757dy.com/). After removing duplicates in Excel 2016, we obtained 298 initial records (160 from GBIF, 88 from CVH, 28 from CNKI, and 22 from Google Scholar). To reduce spatial bias, we applied a 2.5′ × 2.5′ grid in ArcGIS 10.8 and retained the nearest point per grid cell. This procedure yielded 269 refined records (146 from GBIF, 81 from CVH, 22 from CNKI, and 20 from Google Scholar) for modeling (Figure 1). All data were formatted as CSV files following the input requirements of MaxEnt 3.4.4 (http://biodiversityinformatics.amnh.org/open_source/maxent/, accessed on 10 May 2026). The base map of China used in this study was sourced from the Standard Map Service System of the Ministry of Natural Resources of China (http://bzdt.ch.mnr.gov.cn/, accessed on 22 December 2023, map approval number: GS (2023)2762).

### 2.3. Acquisition and Processing of Environmental Variables

In this study, we integrated 24 variables from four categories—climate, topography, vegetation, and human activity—to predict the distribution pattern of *E. sagittatum* (Table S1). The 19 bioclimatic variables [34] and elevation data were obtained from WorldClim version 2.1 (https://www.worldclim.org/, accessed on 8 May 2026). Slope and aspect were subsequently derived from the elevation data using ArcGIS 10.8. The Normalized Difference Vegetation Index (NDVI, from MODIS/Terra MOD13A3) was used to characterize vegetation conditions [35], whereas the Human Footprint Index (HFI) was employed to represent the intensity of human activities [36].

To address multicollinearity among variables, we first extracted the values of all 24 variables at the 269 occurrence points using ArcGIS 10.8. A Spearman correlation analysis (Origin 2026; Figure S1) was then performed for variable screening. Specifically, variables with |r| < 0.7 were retained directly [37]. For groups of variables with |r| ≥ 0.7, only the variable with the highest contribution rate in the initial MaxEnt model (run with all 24 variables) was kept (Table S1). This procedure yielded a final set of 13 variables: Isothermality (Bio3), Maximum Temperature of the Warmest Month (Bio5), Mean Temperature of the Wettest Quarter (Bio8), Mean Temperature of the Driest Quarter (Bio9), Annual Precipitation (Bio12), Precipitation of the Driest Quarter (Bio17), Slope, Aspect, NDVI, and HFI.

Future distribution changes were projected using the BCC-CSM2-MR climate model, which has been shown to perform well for species distribution predictions in China [38]. Projections were made under three Shared Socioeconomic Pathway scenarios (SSP126, SSP370, and SSP585) for three periods: the 2050s (2041–2060), the 2070s (2061–2080), and the 2090s (2081–2100). According to McGregor et al. [39], the radiative forcing levels associated with these scenarios by the year 2100 are approximately 2.6, 7.0, and 8.5 W/m^2^, respectively.

### 2.4. MaxEnt Model Optimization and Prediction

#### 2.4.1. MaxEnt Model Optimization

Default MaxEnt parameters can introduce systematic bias into predicted probabilities [40], potentially overestimating or underestimating the suitable habitat range of *E. sagittatum*. To address this issue, we optimized parameters using the ENMeval 2.0.4 package [31]. Specifically, the regularization multiplier (RM) was adjusted from 0.5 to 4.0 at increments of 0.5. Using five feature types, namely linear (L), quadratic (Q), product (P), threshold (T), and hinge (H), we constructed nine feature combinations (H, HPT, L, LQ, LQH, LQHP, LQHPT, QHP, and QHPT) [41], resulting in 72 candidate models. The optimal model was selected based on the Akaike Information Criterion (AICc) and the 10% training omission rate (OR10) [13]. In general, the model with the lowest AICc value (ΔAICc = 0) and a low OR10 value is considered optimal. When multiple models had AICc values below 2, we further used OR10 to assess predictive conservatism, selecting the model with the smallest OR10 as the final optimal model [42]. This optimal model was then applied to predict the species distribution of *E. sagittatum*.

#### 2.4.2. MaxEnt Model Parameter Settings

Using 269 rigorously quality-controlled distribution records, we developed a MaxEnt modeling workflow that balances accuracy and robustness to predict the potential suitable habitat patterns of *E. sagittatum* under climate change. The specific parameter settings were as follows. Data were partitioned via stratified random sampling into a training set (75%, 202 records) and an independent validation set (25%, 67 records), maintaining structural representativeness of environmental space while ensuring statistical independence. Following current best practices in ecological niche modeling, we used cross-validation for internal calibration, set 10,000 background points to characterize environmental heterogeneity, set the regularization multiplier to 3.5 to avoid overfitting, and selected the QHP feature combination to accurately capture the species’ complex nonlinear responses to environmental factors. The model was run with 10 replicates, and the outputs were integrated to reduce uncertainty from random variation. Finally, we generated logistic-format suitability probability rasters (.asc) for subsequent spatial statistical analysis, multi-scenario comparison, and visualization.

#### 2.4.3. Evaluation of Optimized Model Predictive Accuracy

To assess the predictive reliability of the optimized MaxEnt model, we used three evaluation metrics: the area under the ROC curve (AUC), the True Skill Statistic (TSS), and Cohen’s kappa coefficient (Kappa). AUC reflects the model’s ability to discriminate between presence and background points, with higher values indicating better performance (range: 0.5 to 1). Following the classification scheme of Zhao et al. [43], AUC values were interpreted as follows: 0.5–0.6 (invalid), 0.6–0.7 (poor), 0.7–0.8 (fair), 0.8–0.9 (good), and 0.9–1.0 (excellent). TSS corrects for sample bias by integrating sensitivity and specificity. According to Lu et al. [44], TSS values were classified into five levels: poor (–1 to 0.4), fair (0.4–0.5), good (0.5–0.7), very good (0.7–0.85), and excellent (0.85–1.0). The kappa coefficient evaluates agreement between model predictions and actual distributions after accounting for chance. Following Fielding and Bell [45], kappa values were classified as follows: <0.4 (poor), 0.4–0.75 (good), and >0.75 (excellent).

### 2.5. Classification of Species Suitable Habitats

We used a two-step approach to classify the continuous suitability probability rasters generated by MaxEnt, aiming to establish ecologically meaningful and robust suitable habitat boundaries. In the first step, binarization was performed using the 10th percentile training presence threshold [46]. This threshold allows omission of 10% of training presence points, effectively controlling the false negative rate and filtering out marginal records caused by geocoordinate errors, local microhabitat anomalies, or sampling bias. It strikes a balance between the over-prediction of the minimum presence threshold and the arbitrariness of fixed probability cutoffs. To reduce uncertainty, we used the arithmetic mean of thresholds from 10 repeated runs (0.0992) as the uniform binarization criterion. In the second step, we applied the Natural Breaks (Jenks) method in ArcGIS 10.8 to perform unsupervised classification on the binarized suitability rasters [47]. This method maximizes within-group homogeneity and between-group heterogeneity through iterative computation, objectively identifying intrinsic inflection points in probability value distributions. By integrating the breakpoints identified by the Natural Breaks method with ecological interpretability, we divided the potential suitable habitats of *E. sagittatum* into four ordinal classes: non-suitable (≤0.0992), weakly suitable (0.0992–0.3), moderately suitable (0.3–0.5), and highly suitable (>0.5). This classification system enhances spatial discrimination and provides a spatial stratification basis for assessing suitable habitat dynamics under climate change scenarios and for prioritizing conservation efforts.

### 2.6. Spatial Pattern Changes of Species Suitable Habitats

We assessed changes in the potential suitable habitats of *E. sagittatum* under different future climate scenarios and periods from a spatiotemporal perspective. These changes were classified into three categories: retained (always suitable), lost (currently suitable but unsuitable in the future), and expanded (currently unsuitable but suitable in the future). This classification facilitates systematic understanding of species distribution responses to climate change and provides a basis for conservation prioritization. The analytical steps were as follows. First, current and future suitability probability rasters for the 2050s, 2070s, and 2090s under SSP126, SSP370, and SSP585 were imported into ArcGIS 10.8 and binarized using the 10th percentile training presence threshold (0.0992), generating binary distribution maps where retained areas were coded as 1 → 1, lost areas as 1 → 0, and expanded areas as 0 → 1. Second, the binary rasters were converted to vector polygon layers and overlaid using the Intersect tool, ultimately producing maps of spatial pattern changes in suitable habitats for *E. sagittatum* under each climate scenario.

### 2.7. Centroid Migration of Species Suitable Habitats

We extracted the centroids of suitable habitats for *E. sagittatum* using ArcGIS 10.8 and quantified their spatial migration distances. This analysis covered the current period (1970–2000) and three future periods (2050s, 2070s, 2090s) under three climate scenarios (SSP126, SSP370, and SSP585). The procedure involved three steps: first, the suitability probability rasters generated by MaxEnt were binarized and vectorized using a threshold of 0.0992; second, centroid coordinates for suitable habitat polygons were calculated for each period and scenario using the geometric statistics module; and third, migration distances were calculated using the Euclidean distance formula to reveal the potential spatial displacement characteristics of the species in response to climate change.

## 3. Results

### 3.1. MaxEnt Model Optimization and Accuracy Assessment

**Figure 2:**
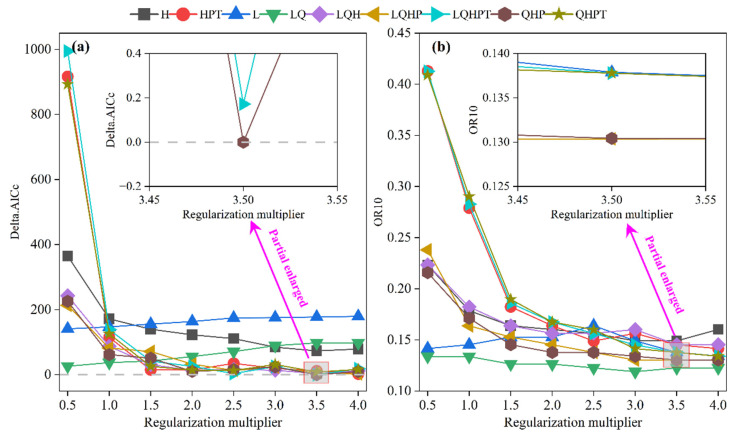
ENMeval-based model selection for *E. sagittatum*: (**a**) ΔAICc values across parameter combinations; (**b**) 10% training omission rates (OR10) for selected models. Feature classes: L (Linear), Q (Quadratic), H (Hinge), P (Product), T (Threshold). Horizontal grey dashed line marks delta AICc = 0 reference.

The default MaxEnt configuration (RM = 1, FC = LQHP) produced a Delta AICc of 82.16, well above the recommended threshold of 2 proposed by Anderson and Gonzalez [48]. This indicates inappropriate model complexity, manifesting as severe overfitting or redundant parameters. Using ENMeval with 269 occurrence records and 13 environmental variables, we identified two candidate models with Delta AICc below 2: RM = 3.5/FC = QHP (Delta AICc = 0) and RM = 3.5/FC = LQHPT (Delta AICc = 0.17) (Figure 2a). To differentiate between these statistically equivalent models, we compared their OR10 values as a measure of predictive conservatism. The OR10 for RM = 3.5/FC = QHP (0.1304) was 5.34% lower than that for RM = 3.5/FC = LQHPT (0.1378) and 20.37% lower than that of the default model (0.1637) (Figure 2b). Thus, the RM = 3.5/FC = QHP model achieves a lower false positive rate and greater predictive conservatism. Based on its Delta AICc optimality (Δ = 0), OR10 advantage, and model parsimony, we selected RM = 3.5/FC = QHP as the optimal MaxEnt parameter configuration.

**Figure 3:**
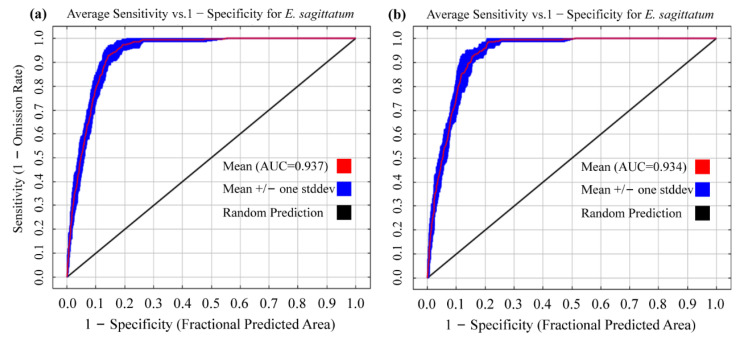
ROC curves and AUC values for *E. sagittatum* from MaxEnt modeling under default parameters (**a**) and after parameter optimization (**b**).

To validate the effectiveness of parameter optimization, we compared the predictive performance of the default model (RM = 1, FC = LQHP) and the optimal model (RM = 3.5, FC = QHP). Both models were run with 10 replicates of cross-validation. The default model achieved an AUC of 0.937 (Figure 3a), whereas the optimal model achieved an AUC of 0.934 (Figure 3b). Both values fall well within the excellent range (AUC ≥ 0.9). The optimal model’s AUC decreased by only 0.003, a reduction far smaller than the typical random fluctuation of AUC across repeated runs (usually ±0.01). In contrast, the ΔAICc between the two models was 82.16. These results demonstrate that the optimized model substantially reduced model complexity and effectively mitigated overfitting risk with almost no loss in discriminative ability. Further accuracy validation showed that the optimized model achieved a True Skill Statistic (TSS) of 0.790, which according to standard criteria is classified as very good (>0.7). The Kappa coefficient was 0.454, indicating moderate agreement between predictions and observations. Taken together with the substantial improvement in OR10, these findings confirm that the optimal parameter combination (RM = 3.5, FC = QHP) maintains excellent predictive accuracy while markedly improving model generalization capacity and ecological interpretability.

### 3.2. Dominant Factors Influencing the Distribution of E. sagittatum

**Figure 4:**
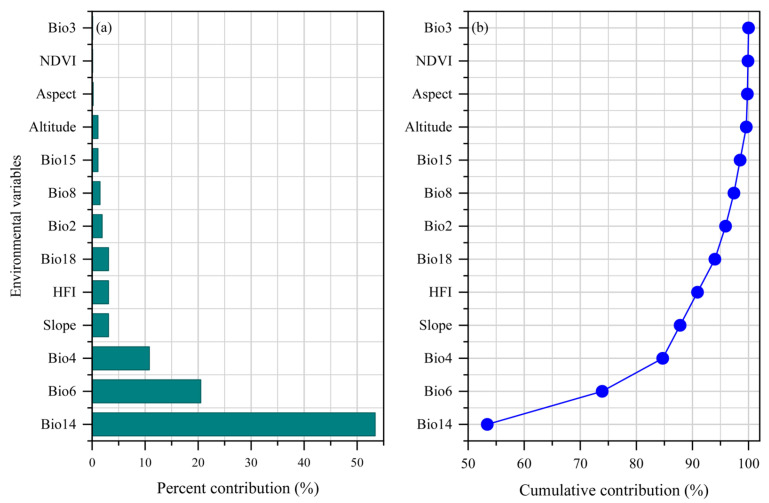
MaxEnt-based contribution percentages of environmental variables to the predicted distribution of *E. sagittatum*. (**a**) the individual contribution percentages of each environmental variable, and (**b**) the cumulative contribution curves of the top-ranking variables.

Using the optimized MaxEnt model, we evaluated the contribution rates of 13 environmental factors to the potential geographic distribution of *E. sagittatum*. As shown in Figure 4, the variables ranked by contribution rate from highest to lowest were as follows: Precipitation of Driest Month (Bio14, 53.4%), Minimum Temperature of Coldest Month (Bio6, 20.5%), Standard Deviation of Temperature Seasonality (Bio4, 10.8%), Slope (3.1%), Human Footprint Index (HFI, 3.1%), Precipitation of Warmest Quarter (Bio18, 3.1%), Mean Diurnal Range (Bio2, 1.9%), Mean Temperature of Wettest Quarter (Bio8, 1.5%), Precipitation Seasonality (Bio15, 1.1%), Altitude (1.1%), Aspect (0.2%), NDVI (0.1%), and Isothermality (Bio3, 0.1%). When aggregated by factor category, climatic factors contributed 92.4% of the total explanatory power, followed by topographic factors (4.4%), human activity (3.1%), and vegetation (0.1%). Applying the conventional screening criterion of cumulative contribution exceeding 85%, the four variables Bio14, Bio6, Bio4, and Slope together explained 87.8% of the variation in suitable distribution. Thus, these four factors overwhelmingly dominate the geographic distribution pattern of *E. sagittatum* in China and constitute the core drivers regulating its potential distribution.

**Figure 5:**
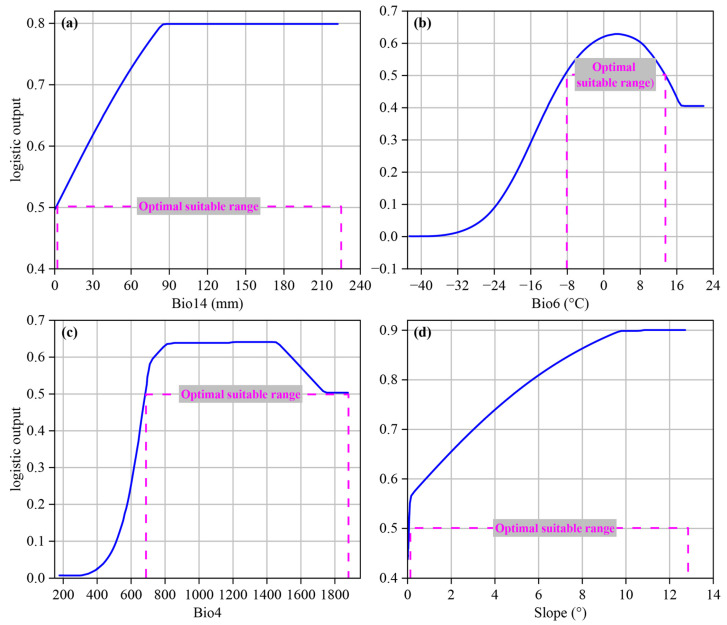
Response curves of the predicted occurrence probability of *E. sagittatum* to the four dominant environmental variables. The blue lines represent the mean values of 10 replicate runs, and the purple dashed lines indicate the optimal ranges. (**a**) Precipitation of the Driest Month (Bio14), (**b**) Minimum Temperature of the Coldest Month (Bio6), (**c**) Standard Deviation of Temperature Seasonality (Bio4), (**d**) Slope.

To explore the relationship between occurrence probability and the four dominant factors, we analyzed marginal effects using univariate response curves (Figure 5). Precipitation of the Driest Quarter (Bio14) showed a positive correlation with occurrence probability (Figure 5a). At 0 mm, the probability was 0.497, rising steadily to a plateau at 85.06 mm (approximately 0.799). Minimum Temperature of the Coldest Month (Bio6) exhibited an asymmetric unimodal response (Figure 5b). The probability approached zero below −30.41 °C, peaked between −3.19 °C and 2.50 °C (approximately 0.629), and then gradually declined. Temperature Seasonality (Bio4) showed a pattern of low-value suppression, rapid mid-value increase, and high-value decline (Figure 5c). The probability was very low (<0.01) when Bio4 fell below 306.03, climbed rapidly to 0.639 between 434.25 and 854.54, remained on a plateau (0.639–0.641) across a broad range (875.91–1452.91), and dropped to approximately 0.5 when Bio4 exceeded 1730.72. Slope showed a positive, saturating relationship (Figure 5d). The probability rose rapidly from 0.446 at 0.02° to 0.899 at 9.79°, then entered a stable plateau with a maximum of 0.900. Integrating these response characteristics with the optimal suitability threshold (occurrence probability ≥ 0.5), the most suitable environmental conditions for *E. sagittatum* are as follows: Precipitation of the Driest Quarter (Bio14) ≥ 1.58 mm, Minimum Temperature of the Coldest Month (Bio6) between −8.34 °C and 13.61 °C, Temperature Seasonality (Bio4) ≥ 658.32, and Slope ≥ 0.06°.

### 3.3. Potential Suitable Habitat Distribution of E. sagittatum Under Current Climate Conditions

**Figure 6:**
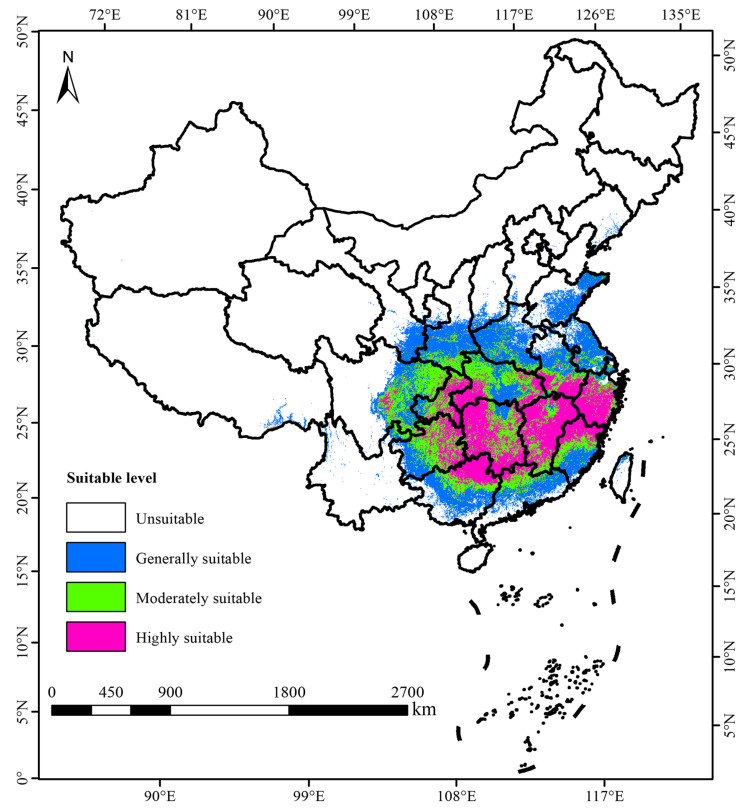
Current potential distribution of *E. sagittatum* in China.

Under current climate conditions, the total suitable habitat for *E. sagittatum* covers approximately 200.89 × 10^4^ km^2^, with weakly, moderately, and highly suitable areas accounting for 80.64 × 10^4^ km^2^ (40.1%), 57.37 × 10^4^ km^2^ (28.6%), and 62.88 × 10^4^ km^2^ (31.3%), respectively. Weakly suitable areas are broadly distributed across southern Henan, Shandong, Shaanxi, Shanxi, Gansu, and Jiangsu, as well as central Anhui and Hubei, northern Hunan, southwestern Guizhou, southern Guangxi, Guangdong, and Fujian, southeastern Tibet, eastern Sichuan, and northern Taiwan. Moderately suitable areas are concentrated in eastern Sichuan, southern Shaanxi, central Chongqing, northern Hubei, central Hunan, southern Henan, central Anhui, southern Jiangsu, and the central parts of Guizhou, Guangxi, Guangdong, Fujian, and Jiangxi. Highly suitable areas are mainly found in Jiangxi, Zhejiang, central Sichuan, eastern Chongqing, eastern and western Hubei, southern Anhui, eastern Guizhou, western and southern Hunan, and the northern parts of Guangxi, Guangdong, and Fujian (Figure 6).

### 3.4. Potential Suitable Habitat Dynamics of E. sagittatum Under Future Climate Change

**Figure 7:**
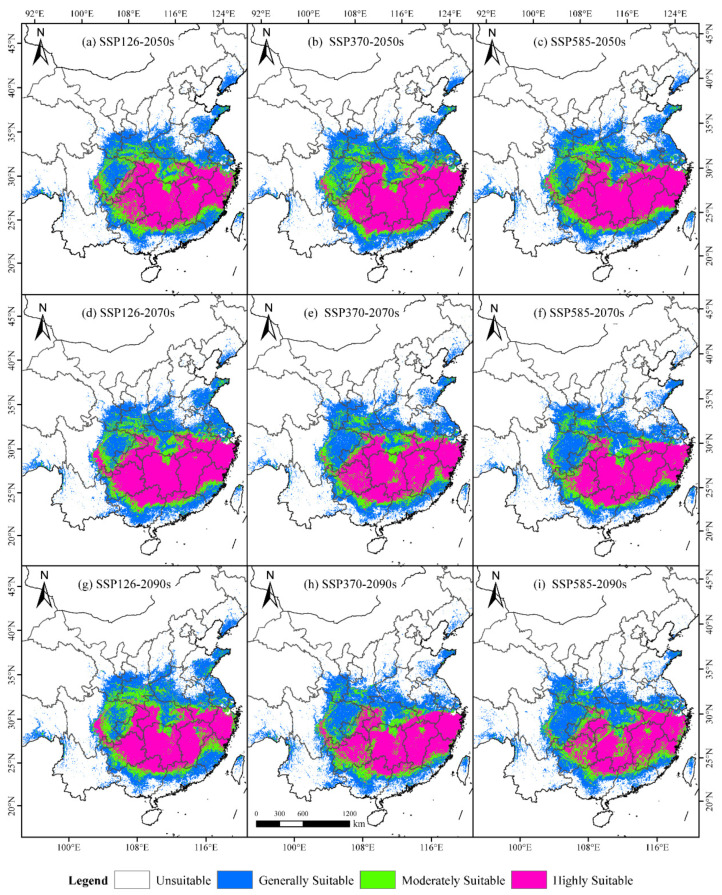
Future suitable habitats for *E. sagittatum* in China under SSP126, SSP370, and SSP585 during the 2050s, 2070s, and 2090s. (**a**) SSP126-2050s, (**b**) SSP370-2050s, (**c**) SSP585-2050s, (**d**) SSP126-2070s, (**e**) SSP370-2070s, (**f**) SSP585-2070s, (**g**) SSP126-2090s, (**h**) SSP370-2090s, and (**i**) SSP585-2090s.

Under the SSP126 scenario (Figure 7a,d,g), the total suitable area remains generally stable with a slight increase across periods: 205.20 × 10^4^ km^2^ in the 2050s, peaking at 214.68 × 10^4^ km^2^ in the 2070s, then slightly declining to 212.80 × 10^4^ km^2^ in the 2090s. The highly suitable area follows a similar trajectory (79.47 → 92.51 → 80.60 × 10^4^ km^2^), initially expanding to include parts of Yunnan, Henan, and Guangxi and then contracting with a moderate northwestward shift.

Under the SSP370 scenario (Figure 7b,e,h), the total area shows a continuous decline from 211.00 × 10^4^ km^2^ (2050s) to 202.59 × 10^4^ km^2^ (2070s) and 202.12 × 10^4^ km^2^ (2090s). The highly suitable area (84.88 → 79.03 → 77.52 × 10^4^ km^2^) exhibits notable middle-period expansion to its northernmost boundary—extending into Jiangsu, southern Shaanxi, and central Anhui—followed by marked contraction.

Under the SSP585 scenario (Figure 7c,f,i), both total and highly suitable areas decline most sharply, from 211.90 × 10^4^ km^2^ to 192.34 × 10^4^ km^2^ and from 82.91 × 10^4^ km^2^ to 66.96 × 10^4^ km^2^, respectively—the latter being the lowest across all scenarios. The highly suitable range becomes progressively fragmented and retreats southward, with notable losses in Hubei, Anhui, and Fujian, and only small pockets retained in southern Shaanxi by the 2090s, indicating strongly negative effects of high emissions on habitat suitability.

### 3.5. Spatiotemporal Dynamics of Suitable Habitats for E. sagittatum Under Future Climate Scenarios

**Figure 8:**
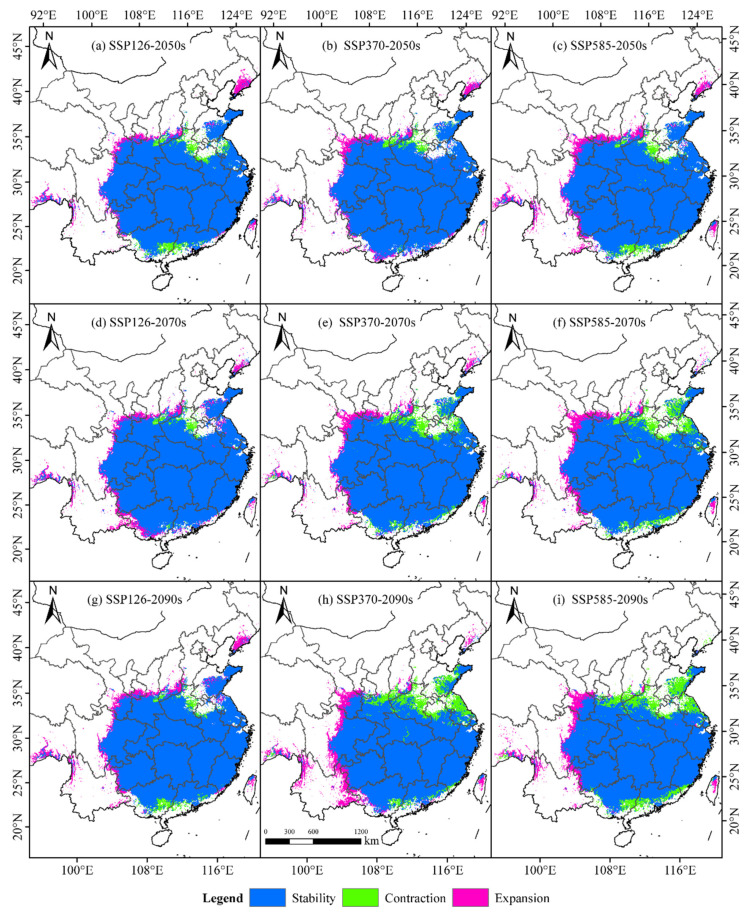
Climate-driven habitat shifts (stable, lost, gained) for *E. sagittatum* under SSP126, SSP370, and SSP585 during the 2050s, 2070s, and 2090s.

Under SSP126 scenario, habitat remains stable with slight net expansion. In the 2050s, stable area is 190.57 × 10^4^ km^2^ (89.24%), with 9.46 × 10^4^ km^2^ (4.43%) lost and 13.52 × 10^4^ km^2^ (6.33%) gained—losses in southern Guangxi, Guangdong, central Shaanxi, southern Shanxi, eastern Henan, and northern Anhui; gains in southeastern Tibet, northwestern Yunnan, central Sichuan, southern Gansu, Ningxia, southeastern Shanxi, northern Taiwan, and central Shaanxi (Figure 8a). In the 2070s, stable area rises to 195.60 × 10^4^ km^2^ (89.65%), losses shrink to 4.43 × 10^4^ km^2^ (2.03%), gains grow to 18.15 × 10^4^ km^2^ (8.32%), extending to northern Jiangsu, central Shandong, and southwestern Guangxi (Figure 8d). In the 2090s, stable area is 193.35 × 10^4^ km^2^ (88.68%), losses at 6.69 × 10^4^ km^2^ (3.07%), gains at 18.00 × 10^4^ km^2^ (8.25%), stabilizing across the above regions plus northern Jiangsu and central Shandong (Figure 8g).

Under SSP370 scenario, a clear contraction emerges. In the 2050s, stable area is 194.53 × 10^4^ km^2^ (90.29%), with 5.51 × 10^4^ km^2^ (2.56%) lost and 15.41 × 10^4^ km^2^ (7.15%) gained—losses in eastern Guangxi, central Shaanxi, southwestern Shanxi, northeastern Henan, and central Shandong; gains in southeastern Tibet, northwestern Yunnan, central Sichuan, southern Gansu, Ningxia, southeastern Shanxi, northern Taiwan, southern Guangxi, Guangdong, and central Shaanxi (Figure 8b). In the 2070s, stable area decreases to 185.87 × 10^4^ km^2^ (86.23%), losses expand to 14.16 × 10^4^ km^2^ (6.57%), gains at 15.52 × 10^4^ km^2^ (7.20%)—losses extend to southern Guangxi, Guangdong, southern Shanxi, eastern Henan, northern Anhui, Jiangsu, and central Shandong; gains shrink to southeastern Tibet, Yunnan, central Sichuan, southern Gansu, Ningxia, southeastern Shanxi, northern Taiwan, and central Shaanxi (Figure 8e). In the 2090s, stable area declines to 177.80 × 10^4^ km^2^ (79.95%), losses reach 22.19 × 10^4^ km^2^ (9.98%), gains at 22.41 × 10^4^ km^2^ (10.08%); southern Jiangsu exits the stable zone, gains restricted to southeastern Tibet, Yunnan, central Sichuan, southern Gansu, Ningxia, southeastern Shanxi, and northern Taiwan (Figure 8h).

Under SSP585 scenario, the most dramatic changes occur. In the 2050s, stable area is 191.43 × 10^4^ km^2^ (87.33%), with 8.64 × 10^4^ km^2^ (3.94%) lost and 19.13 × 10^4^ km^2^ (8.73%) gained—losses in southern Guangxi, Guangdong, central Shaanxi, southern Shanxi, eastern Henan, northern Anhui, Jiangsu, and central Shandong; gains in southeastern Tibet, Yunnan, central Sichuan, southern Gansu, Ningxia, southeastern Shanxi, northern Taiwan, and central Shaanxi (Figure 8c). In the 2070s, stable area decreases to 181.94 × 10^4^ km^2^ (84.19%), losses expand to 18.06 × 10^4^ km^2^ (8.36%), gains at 16.10 × 10^4^ km^2^ (7.45%); southern Jiangsu exits, losses extend to central Hubei, gains shrink to southeastern Tibet, Yunnan, central Sichuan, southern Gansu, Ningxia, southeastern Shanxi, northern Taiwan, and central Shaanxi (Figure 8f). In the 2090s, stable area falls to 174.78 × 10^4^ km^2^ (81.15%), losses reach 25.22 × 10^4^ km^2^ (11.71%), gains at 15.39 × 10^4^ km^2^ (7.15%); southern Jiangsu and other eastern coastal areas lost, gains confined to southeastern Tibet, Yunnan, central Sichuan, southern Gansu, Ningxia, southeastern Shanxi, and northern Taiwan (Figure 8i).

### 3.6. Centroid Migration of E. sagittatum Suitable Habitats Under Future Climate Scenarios

**Figure 9:**
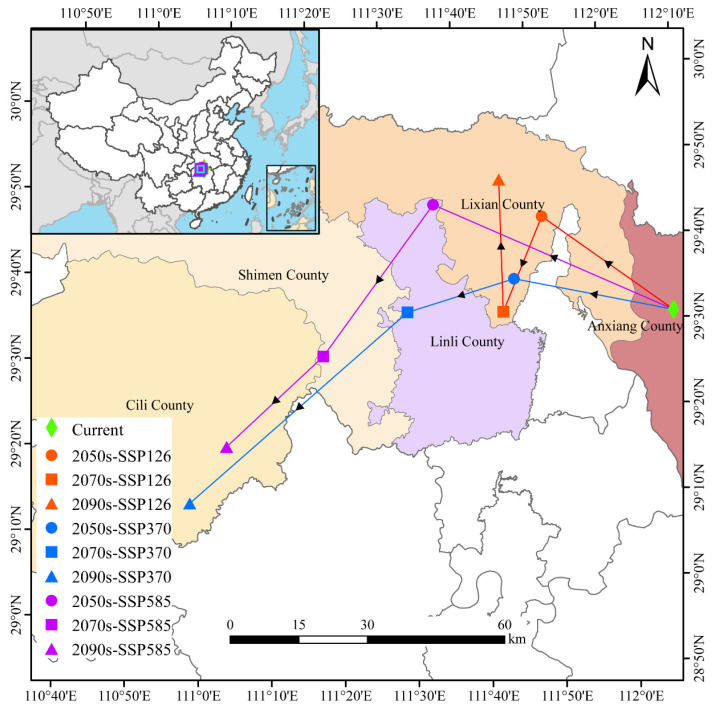
Shifts in centroid of potential habitat for *E. sagittatum* under SSP126, SSP370, and SSP585 during the 2050s, 2070s, and 2090s.

To reveal the spatial displacement patterns of potential suitable habitats for *E. sagittatum* under climate change, we mapped its centroid migration path using ArcGIS 10.8 (Figure 9). Under current climatic conditions (baseline period 1970–2000), the centroid of the natural distribution of *E. sagittatum* is located in Anfeng Township, Anxiang County, Changde City, Hunan Province (112°08′ E, 29°31′ N). Our results show that under all future climate scenarios, the centroids of potential distribution areas exhibit a general westward migration trend. The detailed patterns are as follows.

Under the SSP126 scenario, the centroid migration exhibits a phased westward shift. In the 2050s, the centroid moves approximately 35.32 km northwestward from Anfeng Township to Cennan Town, Lixian County (111°51′ E, 29°43′ N). In the 2070s, it moves approximately 22.39 km southwestward from Cennan Town to Xiumei Town, Linli County (111°45′ E, 29°32′ N). In the 2090s, it moves approximately 28.71 km northwestward from Xiumei Town, finally arriving at Mengxi Town, Lixian County (111°45′ E, 29°47′ N). The cumulative migration distance for this period is approximately 86.42 km, with the overall direction trending northwestward.

Under the SSP370 scenario, the magnitude of centroid migration gradually increases over time. In the 2050s, the centroid moves approximately 35.77 km northwestward from Anfeng Township to Hekou Town, Linli County (111°46′ E, 29°36′ N). In the 2070s, it moves approximately 24.63 km southwestward from Hekou Town to Jiashan Town, Shimen County (111°32′ E, 29°33′ N). In the 2090s, it moves approximately 63.50 km southwestward from Jiashan Town, finally arriving at Longtanhe Town, Cili County, Zhangjiajie City (111°00′ E, 29°12′ N). Under this scenario, the centroid migrates generally southwestward, with a cumulative distance of approximately 123.90 km. Notably, the migration distance increases substantially during the final period.

Under the SSP585 scenario, the centroid migration distance is relatively large even in the early period. In the 2050s, the centroid moves approximately 57.59 km northwestward from Anfeng Township to Dayandang Town, Lixian County (111°36′ E, 29°45′ N). In the 2070s, it moves approximately 40.91 km southwestward from Dayandang Town to Guangfuqiao Town, Cili County (111°20′ E, 29°28′ N). In the 2090s, it moves approximately 28.97 km southwestward from Guangfuqiao Town, finally arriving at Erfangping Town, Cili County (111°06′ E, 29°18′ N). Under this scenario, the cumulative migration distance is approximately 127.47 km, with the centroid migrating generally southwestward and the migration distance being largest during the initial period.

## 4. Discussion

### 4.1. Improvement in Prediction Accuracy Through Model Parameter Optimization

The default parameter configuration of the MaxEnt model (regularization multiplier = 1, feature combination = LQHP) carries a serious risk of overfitting [10,49]. The default model yielded a Delta AICc value of 82.16, far exceeding the commonly recommended threshold for model selection (Delta AICc < 2) [48]. Moreover, its 12 features include product features, which are particularly prone to introducing spurious interaction terms. Consequently, they tend to misinterpret spatial noise in the training set as genuine ecological signals [12]. This issue has been confirmed in multiple MaxEnt studies focusing on plants distributed in China. Studies on *Litsea cubeba* (Lour.) Pers., *Wikstroemia indica* (L.) C. A. Mey., and *Rhus chinensis* Mill. all found that default complex parameter settings often lead to overfitting. In contrast, models with optimized parameter combinations perform better in terms of predictive accuracy and ecological interpretability [50,51,52]. Through parameter searching using ENMeval 2.0, we determined that the optimal configuration for predicting the distribution of *E. sagittatum* is a regularization multiplier of 3.5 combined with the QHP feature combination. This configuration reduced the total number of features from 12 to 7. Compared with the default model, the optimized model’s AUC decreased by only 0.003, a reduction far smaller than the typical random fluctuation range (±0.01 to 0.02) across multiple repeated runs. In contrast, the AICc value decreased by 82.16. This indicates that the optimized model substantially reduces model complexity and eliminates overfitting risk with almost no loss in discriminative ability [13,46].

More importantly, the optimized model achieved a 10% training omission rate of 0.1304, which is approximately 20% lower than that of the default model (typically around 0.1638). This reduction demonstrates a significant decrease in the misclassification of spatial noise in the training set, i.e., the false positive rate. Consequently, the reliability of spatial extrapolation predictions is improved [31]. Based on these results, we recommend that studies with 200 to 500 occurrence points perform a grid search of regularization multipliers between 0.5 and 4 using ENMeval 2.0. The optimal model should be selected as the combination with the lowest AICc and a 10% training omission rate not exceeding 0.15 [48,53]. This optimization approach is consistent with the recommendations of developers of mainstream R packages v 4.2.1 such as ENMeval and Kuenm. These developers advocate using AICc to balance model complexity and goodness-of-fit in species distribution modeling, rather than relying solely on AUC as the model selection criterion [31,54].

### 4.2. Formation of the Distribution Pattern Dominated by Hydrothermal Factors

Precipitation of the Driest Quarter (Bio14) and Minimum Temperature of the Coldest Month (Bio6) together explain 73.9% of the variation in the geographic distribution of *E. sagittatum*, thereby validating our first hypothesis. The response curve for Bio14 shows a nearly linear positive correlation, reaching a plateau at 85.06 mm where the occurrence probability exceeds 0.799. This physiological mechanism may be related to the species’ shallow root system and its rapid response to changes in soil water potential [55,56]. Under climate change, even if annual precipitation remains unchanged, increased precipitation seasonality can significantly reduce the fitness of this species [26,57]. The dominance of dry-season water availability over temperature in shaping *E. sagittatum* distribution aligns with studies on tropical understory medicinal species, where seasonal water deficit similarly limits range expansion [58]. By contrast, in temperate and boreal forests, understory species distributions are often more constrained by low winter temperatures and the availability of cold microrefugia [59]. This contrast suggests that the relative importance of hydrothermal drivers varies systematically with latitude.

Minimum Temperature of the Coldest Month (Bio6) exhibits a narrow optimal temperature window for *E. sagittatum*, ranging from −8.34 °C to 13.61 °C. The occurrence probability approaches zero at the low-temperature end, rises rapidly to a peak with increasing temperature, and then gradually declines as temperatures continue to rise. This asymmetric unimodal response reveals a dual mechanism involving low-temperature limitation and heat stress. Low temperatures limit population establishment and maintenance through frost damage mechanisms [60,61]. Conversely, excessively high winter minimum temperatures may exacerbate pathogen overwintering and reproduction pressure [62,63]. This narrow cold-temperature optimum window may be common among Epimedium species and related understory early-spring flowering herbs [26]. Studies on early-spring ephemeral plants in East Asian understory also indicate that the life history strategies of this group are highly dependent on the normal acquisition of winter low-temperature signals [64]. However, such narrow temperature adaptation windows are often masked in coarse-resolution species distribution models based on annual mean temperature. Variables such as annual mean temperature smooth out seasonal extreme temperature signals and consequently fail to identify suitable habitats for species that depend on specific low-temperature conditions [65].

### 4.3. Spatiotemporal Evolution Characteristics of Suitable Habitat Areas for E. sagittatum

Under different emission scenarios, the trajectories of change in the total suitable area for *E. sagittatum* differ markedly. Under the low-emission scenario (SSP126), the total suitable area fluctuates only slightly (variation < 5%). The highly suitable area initially expands and then contracts but remains generally stable, suggesting that moderate warming can alleviate the low-temperature limitation imposed by Bio6 without destroying core habitats [27,28]. The situation differs under the high-emission scenario (SSP585). The total suitable area expands by 5.48% in the 2050s compared with the current period but then contracts continuously, ultimately declining by approximately 4.26% by the 2090s. This pattern of initial expansion followed by contraction reflects the nonlinear effects of climate change. Early warming favors suitable area expansion, but continued warming combined with intensified drought [66,67] ultimately results in a net loss of habitat suitability [68,69]. A notable contrast is that the absolute area of highly suitable habitat actually increases from 62.88 × 10^4^ km^2^ (current) to 66.96 × 10^4^ km^2^ by the 2090s, representing a net increase of approximately 6.5%. This phenomenon does not contradict the declining trend in total suitable area. The underlying mechanism may be spatial reorganization of highly suitable habitats, where expansion in western mountainous areas exceeds contraction along the eastern coast of East China [68,70].

From a spatial pattern perspective, the reorganization of highly suitable habitats shows three clear directions. First, the hilly basins of Jiangxi, Zhejiang, and eastern Hunan constitute a climate buffer core for this species. This region features complex topography, which provides critical refuge for sensitive species during heatwave events [71,72]. Notably, mid-latitude mountains have historically played similar climate-buffering roles [73]. Second, suitable habitats in southern Guangxi and southern Guangdong are completely lost under the high-emission scenario. Drought stress inhibits protective enzyme activity in *E. sagittatum* and exacerbates membrane lipid peroxidation damage [66]. Furthermore, the synergistic negative effect of combined high-temperature and drought stress far exceeds that of either factor alone [67]. As the frequency of extreme high-temperature events continues to rise along the South China coast [74], the survival pressure on populations in this region will further intensify. Third, under low- and medium-emission scenarios, new suitable patches appear from southeastern Tibet to southern Gansu. This expansion is mainly attributable to a moderate increase in Bio6, which alleviates the long-term limitation imposed by extreme low temperatures on distribution [26,64].

These scenario differences and spatial reorganization phenomena offer two theoretical insights. First, the stability of core habitats under the low-emission scenario suggests that emission reduction policies can translate directly into biodiversity benefits. Second, the coexistence of extinction risk for South China coastal populations under the high-emission scenario and the continued suitability of the Jiangnan hilly buffer core indicates that different geographic populations of the same species face markedly different climate change exposures. This finding therefore calls for differentiated management strategies. Previous studies have also found that species that have long inhabited climatically stable regions often struggle to adapt to rapid warming [1]. Moreover, some Epimedium species exhibit similar patterns of dramatic spatial reorganization with relatively stable total areas [26,27]. These observations suggest that conservationists should pay more attention to the actual value of core areas and the habitat quality of migration zones, rather than merely pursuing numerical changes in suitable area. The southwestward centroid shift (127.47 km under SSP585) diverges from the prevailing poleward and upward trends reported in global syntheses for terrestrial species across Europe, North America, and other temperate regions [1,3]. This divergence indicates that regional water balance may override latitudinal temperature gradients for subtropical monsoon-affected species, a pattern less frequently documented in temperate-zone studies.

### 4.4. Southwestward Migration Pattern of the Suitable Habitat Centroid

This study reveals that the centroid of suitable habitats for *E. sagittatum* shows a significant southwestward migration trend and weak latitudinal displacement, rather than the classic predicted northward migration pattern [2]. The fundamental reason for this discrepancy lies in the subtropical region of eastern China, where the north-south thermal gradient has been superimposed and modified by the east-west precipitation gradient and complex topography [75]. Although the North China Plain experiences significant winter warming, intensified precipitation seasonality results in a soil moisture deficit period of up to 5–6 months during the driest quarter. This exceeds the physiological tolerance threshold of the species, as occurrence probability drops sharply below 0.5 when Bio14 falls below 1.58 mm. Consequently, this deficit acts as a critical ecological filter blocking northward expansion. Recent studies consistently indicate that water availability is the core limiting factor restricting northward expansion of subtropical plants in China [76]. Furthermore, drought stress can effectively offset the thermal suitability benefits brought by climate warming [77,78]. Therefore, future suitable habitat migration of *E. sagittatum* will be dominated primarily by water availability rather than temperature. This finding revises the simplified expectation that warming inevitably leads to northward expansion.

The southwestward migration of the suitable habitat centroid of *E. sagittatum* is primarily driven by the hydrothermal compensation effect of the western mountains. The elevation gradient from the eastern Sichuan Basin to the Qinling-Daba Mountains maintains stable soil moisture by reducing summer evapotranspiration stress, while moderate topography (occurrence probability > 0.90 when slope > 9.79°) enhances local water availability. In addition, south-facing slopes receive increased winter solar radiation, which elevates the minimum temperature of the coldest month from the stressful range in the northern plains (<−8.34 °C) into the optimal window (−8.34 °C to 13.61 °C). This mechanism is common among subtropical montane plants, where the realized niche shaped by local topography often contradicts macroclimate-based predictions [79]. Studies on Litsea cubeba, Wikstroemia indica, and related Epimedium species all support a southwestward rather than northward migration pattern [30,50,51]. Notably, this study reveals a phased southwestward shift in the centroid migration of *E. sagittatum*: under the SSP585 scenario, the migration distance is largest in the early period (2050s: 57.59 km) and then decelerates, suggesting that the expansion potential on the western edge of suitable habitats under the high-emission pathway will be largely realized by the middle of this century.

### 4.5. Three-Tier Planning Recommendations for Conservation and Cultivation

Based on the above predictions of suitable habitat dynamics and centroid migration patterns, we propose a three-tier dynamic planning framework for the conservation of germplasm resources and artificial cultivation of *E. sagittatum*. The first tier is priority in situ conservation. The Jiangnan hilly buffer core (Jiangxi, Zhejiang, and western and southern Hunan) represents the most stable highly suitable area for *E. sagittatum* under both current and future climate scenarios. Therefore, it should be designated as a priority in situ conservation unit. This region features complex topography and high canopy cover, which can mitigate the stress of extreme climate events through local climate buffering mechanisms. We recommend establishing a network of provincial nature reserves in this region, with a focus on controlling excessive reclamation and uncontrolled harvesting of understory cash crops. Existing practice has demonstrated that effective coverage of nature reserve networks is key to ensuring the in-situ conservation of medicinal plants [18]. Furthermore, community-based co-management models have achieved initial success in the conservation of Epimedium species [20]. Specific measures include designating core protection plots where harvesting is prohibited, implementing rotational harvesting and artificial replanting systems in buffer zones, and establishing long-term population monitoring systems.

The second tier is climate-smart introduction. We recommend developing forest–medicinal compound systems at elevations of 800–1200 m in three priority regions: central Sichuan Province (Ya’an to Leshan), eastern Chongqing Municipality (Shizhu to Youyang), and southern Shaanxi Province (Ankang to Hanzhong) [80]. These areas lie on the western edge of the current suitable range. Benefiting from the mountain hydrothermal compensation effect, they exhibit high stability and expansion potential under future climate scenarios. The climate-smart agriculture concept emphasizes maintaining production efficiency while adapting to climate change. Its core strategy is to prioritize introduction areas within suitable habitats that remain stable under future climate scenarios [81]. Forest–medicinal compound systems have been shown to achieve large-scale medicinal material production without destroying native vegetation, making them particularly suitable for the sustainable utilization of understory medicinal plants [22]. Conversely, new cultivation base investments should be avoided in areas predicted to lose suitable habitat after 2050 under the high-emission scenario, such as southern Guangxi and southern Guangdong, to prevent long-term asset stranding risks. It should also be noted that the northwestern expansion areas, from southern Gansu to southeastern Tibet, are currently suitable only for long-term experimental introduction due to high patch fragmentation and poor connectivity. Therefore, these areas should not yet be included in large-scale cultivation base planning.

The third tier is assisted migration and germplasm bank construction. For populations expected to face high extinction risk by the end of this century under the high-emission pathway, we recommend prioritizing assisted migration feasibility assessments. These assessments should include precise predictions of future climate suitability at introduction sites and comprehensive testing of the genetic representativeness of source populations. Only after passing these assessments should assisted migration trials be initiated [82]. We also recommend simultaneously collecting seed materials from these high-risk populations to establish a germplasm resource bank that captures their genetic diversity. This bank should be maintained at the Kunming Institute of Botany and the Zhejiang Germplasm Bank, following established conservation standards for similar rare medicinal plants. Establishing a whole-genome germplasm bank would provide strategic resource reserves for the genetic improvement of medicinal material quality [83,84], thereby mitigating the risk of genetic diversity loss under the worst-case scenario. Finally, assisted migration should be combined with ex situ conservation to form a three-tier integrated system: in situ conservation, near-site cultivation, and ex situ preservation.

### 4.6. Study Limitations and Future Directions

We recognize two limitations. First, rigorous cost-benefit monetization of the proposed strategies, including management costs, cultivation returns, and opportunity costs of inaction, is not feasible with currently available data, although our three-tier framework provides qualitative benefit-risk justifications by matching conservation intensity to climate risk gradients. Formal quantification of these trade-offs requires integration of socioeconomic field surveys with our habitat projections, and we explicitly identify this as a priority for future research. This limitation does not undermine the ecological validity of our projections, but it does mean that practical implementation of the framework would benefit from such complementary data to translate spatial predictions into actionable management plans with clear cost-benefit rationales. Second, we acknowledge the lack of publicly available data on commercial value, cultivation extent, and market dynamics of E. sagittatum. Future research should integrate socioeconomic surveys with our habitat projections to enable cost-benefit assessments for conservation prioritization and cultivation site selection.

## 5. Conclusions

This study confirms that parameter optimization of the MaxEnt model is critical for assessing the responses of medicinal plants to climate change. The optimized model for *E. sagittatum* (RM = 3.5, FC = QHP) maintains high discriminative accuracy while significantly improving spatial extrapolation reliability. From the perspective of ecological driving mechanisms, Precipitation of the Driest Quarter (Bio14) and Minimum Temperature of the Coldest Month (Bio6) constitute a dual hydrothermal filtering system. Water deficit during the dry season blocks northward expansion, whereas winter low temperatures limit the establishment of high-latitude populations. Western mountains create locally suitable microenvironments through elevation and aspect modulation. This finding revises the simplified expectation that warming inevitably leads to northward expansion and indicates that water availability will become a key driver of distributional reorganization for subtropical understory plants. We propose a three-tier management strategy. First, the Jiangnan hills, as the climate buffer core, should be prioritized for in situ conservation. Second, the mountains of Sichuan, Chongqing, and southern Shaanxi should be developed for forest–medicinal compound systems. Third, high-risk populations along the South China coast should initiate assisted migration assessments and long-term germplasm preservation. The fundamental stability of core habitats under the low-emission scenario suggests that emission reduction policies can yield direct biodiversity conservation benefits.

## Data Availability

The original contributions presented in this study are included in this article. Further inquiries can be directed to the corresponding authors.

## Supplementary Materials

The following supporting information can be downloaded at: https://www.mdpi.com/article/10.3390/biology15141103/s1, Figure S1: Spearman correlation matrix of the 24 environmental variables. Ellipse elongation reflects the strength of the absolute correlation coefficient: the narrower the ellipse, the stronger the absolute correlation. Color intensity indicates the direction of correlation, with darker blue representing stronger negative correlation and darker red representing stronger positive correlation; Table S1: Twenty-four environmental variables used in this study.
