# Supplementary material for: Climate-Driven Range Shift of the Medicinal Herb Epimedium sagittatum: An Optimized MaxEnt Projection for China
Source: Biology (Basel). 2026 Jul 8;15(14):1103. doi: 10.3390/biology15141103 (PMC13405981; doi:10.3390/biology15141103)
Supplement: Supplementary file 1 [file biology-15-01103-s001.zip › biology-4410883-supplementary.pdf]

Supplementary materials

Climate-Driven Range Shift of the Medicinal Herb *Epimedium sagittatum*: An Optimized MaxEnt Projection for China

Jun Luo [1], Suhang Li [2], Fuyuan Huang [1], Qiong Yang [2], Yangzhou Xiang [2,*], and Ying Liu [3,*]

[1] Guizhou University of Traditional Chinese Medicine, Guiyang 550025, China

[2] School of Geography and Resources, Guizhou Education University, Guiyang 550018, China

[3] School of Biological Sciences, Guizhou Education University, Guiyang 550018, China

Correspondence: yzhxiang18@126.com (Y.X.); lyrainye@126.com (Y.L.).

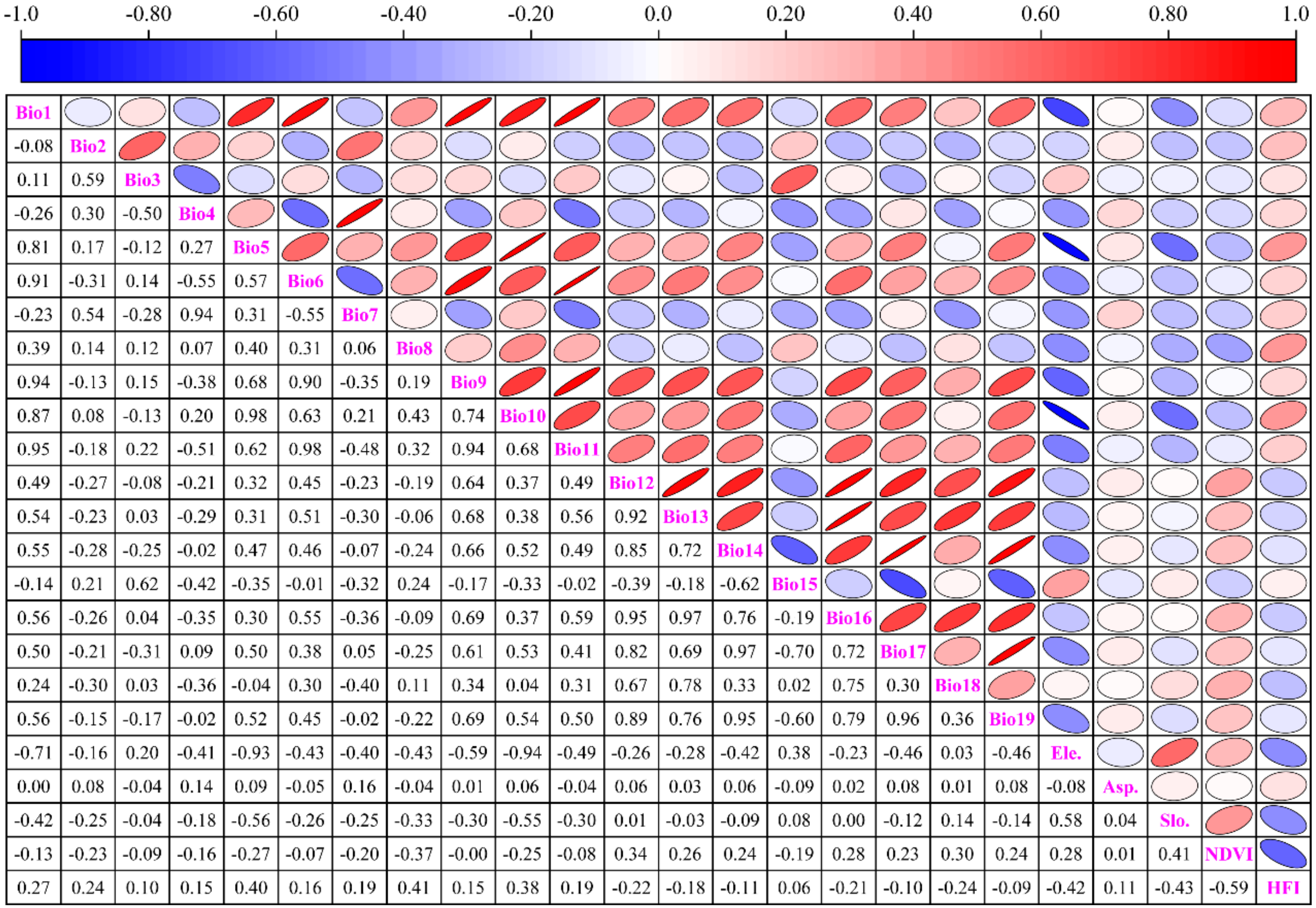

Figure S1. Spearman correlation matrix of the 24 environmental variables. Ellipse elongation reflects the strength of the absolute correlation coefficient: the narrower the ellipse, the stronger the absolute correlation. Color intensity indicates the direction of correlation, with darker blue representing stronger negative correlation and darker red representing stronger positive correlation.

Table S1. Twenty-four environmental variables used in this study.

Category	Abbreviation	Environmental variables	Units	Range	Percent contribution	Weather for Modeling
Bioclimatic	Bio1	Annual mean temperature	°C	1.84 – 22.43	0.2	×
	**Bio2**	**Mean diurnal range (Mean of monthly)**	**°C**	**6.34 – 14.35**	**1.2**	√
	**Bio3**	**Isothermality (Bio2/Bio7) (× 100)**		**23.07 – 40.22**	**0.5**	√
	**Bio4**	**Standard deviation of temperature seasonality**		**573.35 – 1076.05**	**12**	√
	Bio5	Max temperature of warmest month	°C	17.00 – 34.29	0.3	×
	**Bio6**	**Min temperature of coldest month**	**°C**	**-18.67 – 9.72**	**9.4**	√
	Bio7	Temperature annual range (Bio5-Bio6)	°C	23.27 – 39.50	1	×
	**Bio8**	**Mean temperature of wettest quarter**	**°C**	**9.61 – 27.76**	**1.3**	√
	Bio9	Mean temperature of driest quarter	°C	-7.46 – 17.14	0.3	×
	Bio10	Mean temperature of warmest quarter	°C	10.22 – 28.75	0.3	×
	Bio11	Mean temperature of coldest quarter	°C	-7.46 – 14.74	0.5	×
	Bio12	Annual precipitation	mm	553.00 – 2067.00	16.4	×
	Bio13	Precipitation of wettest month	mm	119.00 – 400.00	0	×
	**Bio14**	**Precipitation of driest month**	**mm**	**2.00 – 53.00**	**44.9**	√
	**Bio15**	**Variation of precipitation seasonality**		**45.67 – 114.31**	**1.8**	√
	Bio16	Precipitation of wettest quarter	mm	321.00 – 958.00	0.6	×
	Bio17	Precipitation of driest quarter	mm	12.00 – 205.00	0.1	×
	**Bio18**	**Precipitation of warmest quarter**	**mm**	**283.00 – 943.00**	**0.3**	√
	Bio19	Precipitation of coldest quarter	mm	12.00 – 248.00	0.3	×
Topographic	**Elevation**	**Elevation**	**m**	**1.00 – 3463.00**	**2.5**	√
	**Aspect**	**Aspect**	°	**2.44 – 359.20**	**0.3**	√
	**Slope**	**Slope**	°	**0.00 – 6.14**	**2.6**	√
Vegetation	**NDVI**	**Normalized difference vegetation index**		**0.02 – 0.76**	**0.4**	√
Human	**HFI**	**Human footprint index**		**1.14 – 49.92**	**2.6**	√
